# COVID-19-related fear, stress and depression in school principals: impacts of symptoms like COVID-19, information confusion, health-related activity limitations, working hours, sense of coherence and health literacy

**DOI:** 10.1080/07853890.2022.2101688

**Published:** 2022-07-25

**Authors:** Tuyen Van Duong, Minh H. Nguyen, Chih-Feng Lai, Sheng-Chih Chen, Kevin Dadaczynski, Orkan Okan, Cheng-Yu Lin

**Affiliations:** a School of Nutrition and Health Sciences, Taipei Medical University, Taipei, Taiwan; b International Ph.D. Program in Medicine, College of Medicine, Taipei Medical University, Taipei, Taiwan; c Department of Education, National Taichung University of Education, Taichung, Taiwan; d Master's Program of Digital Content and Technologies, College of Communication, National Chengchi University, Taipei, Taiwan; e Public Health Centre Fulda, Fulda University of Applied Sciences, Fulda, Germany; f Center for Applied Health Science, Leuphana University Lueneburg, Lueneburg, Germany; g Department of Sport and Health Sciences, Technical University Munich, Munich, Germany; h Department of Radio, Television & Film, Shih Hsin University, Taipei, Taiwan

**Keywords:** Stress, fear, COVID-19, depression, health-related activity limitations, information confusion, sense of coherence, health literacy, working hours, school principals

## Abstract

**Background:**

School principals have been reported to have a higher prevalence of burnout and psychological problems than their colleagues. During the pandemic, extra workload and pressure from unprecedented situations potentially cause fear, stress and depression. Therefore, we aimed to explore associated factors of stress, fear of COVID-19 (F-CoV-19S) and depressive symptoms among school principals.

**Methods:**

A cross-sectional online survey was conducted in Taiwan from 23 June to 16 July 2021. Data of 413 school principals were collected, including socio-demographic factors, COVID-19-related factors, work-related information, health status, sense of coherence (SoC), health literacy (HL), F-CoV-19S, stress and depression. Multiple linear and logistic regression models were utilized to explore associations.

**Results:**

School principals with symptoms like COVID-19 (S-COVID-19-S), or with health-related activity limitations had a higher score of stress (*B* = 0.92; *p* = .039) (*B* = 1.52; *p* < .001) and a higher depression likelihood (OR = 3.38; *p* < .001) (OR = 3.06; *p* < .001), whereas those with a better SoC had a lower stress score (B = −1.39; *p* < .001) and a lower depression likelihood (OR = 0.76; *p* = .020). School principals confusing about COVID-19-related information had a higher score of stress (*B* = 2.47; *p* < .001) and fear (*B* = 3.77; *p* < .001). The longer working time was associated with a higher fear score (*B* = 1.69; *p* = .006). Additionally, school principals with a higher HL score had a lower stress score (B = −1.76; *p* < .001), a lower fear score (B = −1.85; *p* < .001) and a lower depression likelihood (OR = 0.53; *p* = .043).

**Conclusions:**

Health-related activity limitations, S-COVID-19-S, COVID-19-related information confusion and longer working hours were positively associated with at least one mental health problem (e.g. stress, fear and depression), whereas better SoC and HL showed the benefits to mitigate fear, stress and depressive symptoms in school principals. Our study provides evidence for appropriate strategies to improve principals’ mental health during the pandemic.

**Key messages:**
- School principals with health-related activity limitations or with symptoms like COVID-19 were more likely to be stressed and depressed.
- Higher levels of stress and fear were observed in school principals who confused about COVID-19-related information, and who had longer working time than before the pandemic.
- Better sense of coherence and higher health literacy could potentially mitigate the fear, stress and depressive symptoms in school principals.

## 1. Introduction

Since its appearance at the end of 2019, COVID-19 has spread rapidly worldwide, causing more than six million deaths by May 2022 [1]. Although several vaccines have been developed and distributed worldwide [2,3], the pandemic is still not under control [4,5]. The COVID-19 pandemic has influenced all aspects of life, especially in education [6]. Most countries have applied epidemic prevention measures (e.g. isolation, lockdown and social distancing) on different scales, forcing schools to close and reopen several times. The education programs have been transitioned from physical classes to virtual ones [7,8]. This posed the unprecedented challenges to the education system, and school principals were under pressures.

The school principals are not only responsible for implementing appropriate policies and practical measures to sustain teaching and learning during school closures but also to ensure a safe educational environment when schools reopened [9]. Therefore, the principals had to face more pressure and stress, which may affect their psychological health during the pandemic. In addition, the principals are in charge of managing and coordinating activities of education authorities, teachers, students and their parents, and to improve student’s performances and teacher’s satisfaction. They are also responsible for implementing the educational policies to achieve learning and teaching goals [10]. Due to the heavy job requirements, school leaders are exposed to increased pressure and stress [11,12].

Before the pandemic, many stressors of school heads were reported in previous research, including student’s achievements, time management, connections with supervisors and personnel, finance, conflict resolution, increased tasks, making tough decisions and lack of autonomy [13,14]. During the pandemic, school principals’ responsibilities have changed unexpectedly and considerably. They are expected to be dynamic and flexible in allocating resources and finances, complying with infection prevention guidelines, and motivating and collaborating with teachers and students to ensure learning and teaching activities, even in a virtual environment [15]. The extra work and burden placed school leaders under enormous pressure, which may cause burnout, stress and other mental health problems [11]. In addition, the uncertainty of the pandemic may also increase their concerns and fears about the uncontrolled spread of COVID-19 and the health of themselves, family members and friends if getting the infection. As a result, the increased stress, fear, and even mental disorders experienced by principals during the pandemic could adversely affect their physical health and work performance [13,16,17]. Furthermore, social isolation, lack of social support from management, colleagues and lack of connections with pupils caused by lockdown measures were the main sources of stress and psychological problems during the COVID-19 pandemic [18]. Meanwhile, school principals play an important role in the response and recovery of the education system from the pandemic [19], which puts an extra burden on them. Therefore, their mental health needs more attention to.

Although fear of COVID-19 and other psychological problems have been investigated in the general population [20,21], patients [22–24], healthcare workers [25,26], students [27,28], and teachers [29–31], research on school principals was still limited during the pandemic. One previous study investigated health literacy in principals and its investigation before the COVID-19 pandemic [32]. Besides, information related to COVID-19 was also disseminated rapidly on social platforms with different quality [33,34]. The circulation of fake news and conspiracy theories could make school heads more confused and worried about the pandemic [35,36], putting more pressure on them to maintain their school operations. Furthermore, as the pandemic has caused significant changes in daily life and working conditions, factors related to COVID-19 (e.g. vaccinations, symptoms like COVID-19 (S-COVID-19-S), or working time changes) should be considered in relation to the psychological health of school principals. Previous studies showed that S-COVID-19-S were associated with a higher likelihood of mental disorders in medical staff [26], and outpatients [22,37]. The longer working time was found to be positively associated with stress, depression and suicidal ideation in different populations [38,39]. However, the impacts of health literacy (HL), COVID-19-related information, S-COVID-19-S and working time on fear, stress and depression remain to be investigated in school principals during the pandemic.

Moreover, sense of coherence (SoC) is defined as the ability to deal with everyday stressors [40], which may help school principals to reduce stress, fear and mental problems when facing unexpected situations during the pandemic. Moreover, the prevalence of common psychological disorders, such as depression or anxiety, has been documented to have differences between gender, age, ethnicity, income, education and residential regions [41,42]. For these reasons, SoC and socio-demographic factors should also be investigated as independent variables in the school principal’s study.

Therefore, we conducted an online survey to explore factors associated with stress, fear of COVID-19, and depression among school principals in Taiwan, in which the impacts of S-COVID-19-S, COVID-19-related information, health-related activity limitations, working hours, SoC and health literacy were emphasized.

## 2. Methods

### 2.1. Study design, data collection, and sample size

A cross-sectional online survey was conducted in Taiwan from 23 June to 16 July 2021 as part of the international COVID-HL school principal project [43,44]. School principals from all 3909 schools in Taiwan (including primary schools, junior high schools, senior secondary schools and schools for special children) [45] were invited to participate in the study.

We recruited potential participants using a network sampling method. First, we contacted the Ministry of Education and local government agencies to send the online survey link to the school principals’ Line group (for internal communications between principals). Additionally, we also contacted the National Association of Primary and Secondary Principals to post the survey link on the member Line group (for internal communications between the heads of City or County Association of Primary and Secondary Principals). Then, the heads announced the survey and sent the survey link to their Principals’ Line groups. Participants signed an online informed consent form before taking the survey. Each survey took 25–30 min to complete. No data are missing due to all questions marked with the mandatory answering option.

The sample size of 318 was calculated based on the α level of 0.05, power of 0.95, the effect size of 0.1 and 19 predictors for multiple linear regression using the G Power software version 3.1.9.7 for Windows [46]. In the current study, 413 principals (out of 3909 potential principals, a response rate of 10.5%) completed the survey, which was satisfactory for analysis. Finally, data were cleaned, coded and analysed anonymously.

### 2.2. Measurements

#### 2.2.1. Outcome variables

The level of COVID-19-related fear was evaluated using the fear of COVID-19 scale (FCoV-19S) [47], which was validated and utilized in Taiwan [48–50]. School heads were asked to rate their agreement on seven items with a 5-point Likert scale from “1 = strongly disagree” to “5 = strongly agree”. The overall scores varied between 7 and 35, where principals with a higher score have a higher level of fear.

Stress was evaluated using the 10-item perceived stress scale (PSS-10) [44] that was commonly used in research [51–53]. The instrument was validated and utilized in Taiwanese studies [54,55], and in healthcare workers during the pandemic [56]. School heads were asked to answer each item with five possible responses from “0 = never” to “4 = very often”. Scores of items 4, 5, 7 and 8 have been reversed. Overall scores varied between 0 and 40, where principals with a higher score have a higher level of stress.

Depression was evaluated using the two-item patient health questionnaire (PHQ-2). This is desirable for use in busy clinical settings [57], which is appropriated for quick assessment during the pandemic. The questionnaire was validated and used in Taiwanese studies [58]. The PHQ-2 score ranges from 0–6; a score of ≥ 2 may be preferable to ensure that few cases of depression are missed [59].

#### 2.2.2. Covariates

Socio-demographic characteristics include age, sex, school types (primary school, junior high school, senior high school/vocational school, school for special children), school geography (north, centre, south, east, outlying islands), school size (≤ 12, 13-24, 25-48, or ≥ 49 classes). The classification was based on the regulation of Taiwan Ministry of Education [60].

Working information: School heads were asked if they were involved in teaching, working time every week and changes in working time as compared with that before the pandemic.

Health status: School heads rated their perceived health conditions with five options from “very bad” to “very good”. Then, principals’ responses were regrouped into two categories: “Very bad/bad/moderate” versus “Good/very good”. Next, information regarding chronic medical conditions (none vs. one or more), and health-related activity limitations caused by chronic conditions (not limited vs. limited) was collected.

Symptoms like COVID-19 (S-COVID-19-S) were investigated [61], consisting of fever, cough, dyspnoea, myalgia, fatigue, sputum production, confusion, headache, sore throat, rhinorrhea, chest pain, haemoptysis, diarrhoea and nausea/vomiting. Then, we divided S-COVID-19-S into two categories (no vs. yes).

COVID-19 vaccination: A question was asked to identify the vaccination status “Have you been vaccinated against COVID-19?” with two response options (yes vs. no).

The level of informing with COVID-19-related updates was evaluated using the question “How well informed do you feel about the COVID-19-related information?” with five possible options from “insufficiently informed” to “very well informed”. Then, responses were categorized into “Insufficient/poor/fine informed” versus “Well or very well informed”. In addition, confusion about COVID-19-related information was also assessed using the question “How much confusion do you feel about COVID-19-related information?” with four options from “not at all confused” to “very confused” [62]. Responses then were regrouped into “Not at all or little confused” versus “Quite or very confused”.

The perceived COVID-19 threat was evaluated using 2 questions “How concerned are you that you or a family member could get infected with coronavirus in the next 1 year?” with four options from “very concerned” to “not concerned at all”, and “How likely is it that you or a family member could get infected with coronavirus in the next 1 year?” with four options from “very likely” to “definitely not” [63]. Responses were categorized into “Slightly concerned/not concerned at all” versus “Very concerned/concerned”, and “Not likely/definitely not” versus “Very likely/somewhat likely”, respectively.

Sense of coherence (SoC) was evaluated using the 9-item scale that was developed and validated in a prior study [64]. The original scale focuses on the work-related context (“How do you personally find your current job and work situation in general?”) [64]. This instrument has been adapted to evaluate the general living condition during the pandemic in China [65]. The scores for each item range from 0 to 6, and scores of items 1, 3, 6, 7 and 9 have been reversed to calculate the mean scores, where a higher score illustrates a better SOC [64,65].

This study used the 22-item HLS-COVID-Q22 questionnaire to assess coronavirus-related health literacy (HL). The tool was developed and validated in prior studies in Germany [62], and in Taiwan [66]. This instrument measures the principals’ ability to seek, understand, evaluate and apply health information in the pandemic context [62]. Principals answered each item with four options varying from “1 = very difficult” to “4 = very easy”. The mean scores varied between 1 and 4, where a better score illustrates a better coronavirus-related HL [62].

### 2.3. Statistical analysis

Firstly, the distributions of stress and fear were checked for normality. Stress was normally distributed (Skewness of 0.107, SE = 0.120), and fear of COVID-19 scale was also normally distributed after excluded 10 outliers (Skewness of 0.089, SE = 0.122) [67]. Secondly, the distributions of stress, fear, and proportion of depression were tested using the one-way ANOVA test, and Chi-square test appropriately. Next, linear and logistic regression models were analysed to explore the associated factors of stress, fear and depression appropriately. To address confounders’ residual effects, all factors in bivariate models were added in multivariate models [68]. To address multicollinearity, the relationships between independent factors were analysed using the Spearman correlation. School geography and school size correlated at *rho* = −0.40, health-related activity limitations were correlated with health status at *rho* = −0.43, and chronic health conditions at *rho* = 0.55, confusion about COVID-19-related information was correlated with informing on COVID-19 updates at *rho* = −0.32, concern about getting infected and perceived likelihood of getting infected was correlated at *rho* = 0.80 (Table S1). Therefore, school geography, health-related activity limitations, COVID-19-related information confusion and concern about getting infected were selected to represent other factors to be analysed in multivariate models appropriately. All analyses were processed using IBM SPSS Version 20.0 for Windows (IBM Corp, Armonk, NY, USA). The significance level was set at a *p*-value < .05.

## 3. Results

### 3.1. Participants’ characteristics

The mean age of school principals was 52.8 ± 4.9 years. Out of the study sample, 35.1% were women, 73.1% were not involved in teaching, 17.2% reported a longer working time than before the pandemic, 38.7% had at least one chronic condition, 28.8% had health-related activity limitations, 36.6% had suspected COVID-19 symptoms, 12.3% reported to be vaccinated. Of all, 87.9% were well or very well informed on COVID-19, 9.9% were quite or very confused about the COVID-19-related information, 65.6% were concerned or very concerned about getting infected and 58.1% perceived likely or very likely to getting infected. The mean scores of SoC, Coronavirus-related HL, perceived stress and fear of COVID-19 were 4.2 ± 1.1, 3.2 ± 0.5, 12.7 ± 4.5, 17.1 ± 4.9, respectively. The proportion of depression (PHQ ≥ 2) was 29.3% (Table 1).

**Table 1.** Study participants’ characteristics, stress, fear of COVID-19, and depression (*N* = 413).

Variables	Total (*n* = 413)	Stress (*n* = 413)		FCoV-19S (*n* = 403)		PH*Q* < 2 (*n* = 292)	PH*Q* ≥ 2 (*n* = 121)	
	*n* (%)	Mean ± SD	p*	Mean ± SD	p*	*n* (%)	*n* (%)	p*
Age, mean ± SD	52.8 ± 4.9					53.1 ± 4.8	51.9 ± 4.8	.019
Gender			.188		.692			.306
Women	145 (35.1)	13.0 ± 4.6		17.3 ± 4.7		98 (33.6)	47 (38.8)	
Men	268 (64.9)	12.4 ± 4.5		17.1 ± 5.1		194 (66.4)	74 (61.2)	
School type			.566		.166			.153
Primary school	260 (63.0)	12.6 ± 4.6		17.4 ± 4.9		191 (65.4)	69 (57.0)	
Junior high school	84 (20.3)	13.1 ± 4.4		17.4 ± 4.6		51 (17.5)	33 (27.3)	
Senior high school or vocational school	60 (14.5)	12.4 ± 4.5		15.9 ± 4.8		44 (15.1)	16 (13.2)	
School for special children	9 (2.2)	11.2 ± 5.7		16.1 ± 7.2		6 (2.1)	3 (2.5)	
School geography			.852		.709			.304
North	124 (30.0)	12.3 ± 4.5		17.3 ± 4.8		95 (32.5)	29 (24.0)	
Centre	135 (32.7)	12.6 ± 4.6		17.5 ± 4.9		88 (30.1)	47 (38.8)	
South	84 (20.4)	13.0 ± 4.8		16.6 ± 5.2		57 (19.5)	27 (22.3)	
East	43 (10.4)	12.7 ± 4.3		16.9 ± 4.7		32 (11.0)	11 (9.1)	
Outlying islands	27 (6.5)	13.0 ± 3.9		16.7 ± 5.3		20 (6.8)	7 (5.8)	
School Size			.866		.810			.139
≤ 12 classes	157 (38.0)	12.8 ± 4.6		17.3 ± 4.8		106 (36.3)	51 (42.1)	
13-24 classes	76 (18.4)	12.3 ± 4.4		16.7 ± 4.9		62 (21.2)	14 (11.6)	
25-48 classes	103 (25.0)	12.7 ± 4.4		17.3 ± 4.9		70 (24.0)	33 (27.3)	
≥ 49 classes	77 (18.6)	12.7 ± 4.9		17.0 ± 5.4		54 (18.5)	23 (19.0)	
Involving teaching			.211		.574			.907
No	302 (73.1)	12.8 ± 4.3		17.2 ± 4.9		214 (73.3)	88 (72.7)	
Yes	111 (26.9)	12.2 ± 5.1		16.9 ± 5.1		78 (26.7)	33 (27.3)	
Weekly working hours, mean ± SD	48.8 ± 14.9		.378		.337			.479
< 40 h	34 (8.2)	13.7 ± 4.9		18.3 ± 4.1		21 (7.2)	13 (10.7)	
= 40 h	62 (15.0)	12.5 ± 4.2		16.9 ± 5.6		45 (15.4)	17 (14.0)	
> 40 h	317 (76.8)	12.6 ± 4.5		17.1 ± 4.9		226 (77.4)	91 (75.2)	
Weekly working hours changes			.085		.082			.066
Less than before the pandemic	117 (28.3)	12.7 ± 4.2		17.0 ± 4.9		83 (28.4)	34 (28.1)	
About the same	224 (54.2)	12.3 ± 4.4		16.8 ± 4.9		166 (56.8)	58 (47.9)	
More than before the pandemic	72 (17.2)	13.7 ± 5.4		18.3 ± 4.7		43 (14.7)	29 (24.0)	
Health status			<.001		<.001			<.001
Very bad/bad/moderate	165 (40.0)	13.8 ± 4.6		18.3 ± 5.1		86 (29.5)	79 (65.3)	
Good/very good	248 (60.0)	11.9 ± 4.3		16.4 ± 4.7		206 (70.5)	42 (34.7)	
Chronic health conditions			.395		.148			.002
None	253 (61.3)	12.5 ± 4.4		16.9 ± 5.0		193 (66.1)	60 (49.6)	
One or more	160 (38.7)	12.9 ± 4.7		17.6 ± 4.8		99 (33.9)	61 (50.4)	
Health-related activity limitations			<.001		.001			<.001
Not limited	294 (71.2)	12.1 ± 4.3		16.6 ± 4.8		233 (79.8)	61 (50.4)	
Limited	119 (28.8)	14.0 ± 4.8		18.4 ± 5.0		59 (20.2)	60 (49.6)	
S-COVID-19-S			<.001		.008			<.001
No	262 (63.4)	12.0 ± 4.3		16.7 ± 4.8		210 (71.9)	52 (43.0)	
Yes	151 (36.6)	13.8 ± 4.7		18.0 ± 5.0		82 (28.1)	69 (57.0)	
COVID-19 vaccination			.511		.727			.499
No	362 (87.7)	12.6 ± 4.5		17.1 ± 4.9		258 (88.4)	104 (86.0)	
Yes	51 (12.3)	13.0 ± 4.4		17.4 ± 5.5		34 (11.6)	17 (14.0)	
Level of informing on COVID-19 updates			<.001		<.001			.654
Insufficient/poor/fine informed	50 (12.1)	15.2 ± 4.4		19.5 ± 4.7		34 (11.6)	16 (13.2)	
Well/very well informed	363 (87.9)	12.3 ± 4.4		16.8 ± 4.9		258 (88.4)	105 (86.8)	
Confusion about COVID-19-related information			<.001		<.001			.004
Not at all/little confused	372 (90.1)	12.3 ± 4.3		16.7 ± 4.7		271 (92.8)	101 (83.5)	
Quite/very confused	41 (9.9)	16.1 ± 5.3		21.4 ± 4.9		21 (7.2)	20 (16.5)	
Level of concern about getting infected			.031		<.001			.202
Slightly concerned/not concerned at all	142 (34.4)	12.0 ± 4.4		14.8 ± 4.6		106 (36.3)	36 (29.8)	
Very concerned/concerned	271 (65.6)	13.0 ± 4.6		18.4 ± 4.6		186 (63.7)	85 (70.2)	
Perceived likelihood of getting infected			.192		<.001			.556
Not likely/definitely not	173 (41.9)	12.3 ± 4.3		15.5 ± 4.8		125 (42.8)	48 (39.7)	
Very likely/somewhat likely	240 (58.1)	12.9 ± 4.7		18.4 ± 4.7		167 (57.2)	73 (60.3)	
Sense of coherence, mean ± SD	4.2 ± 1.1	–	–	–	–	4.3 ± 1.1	3.9 ± 1.0	.001
Coronavirus-related HL, mean ± SD	3.2 ± 0.5	–	–	–	–	3.3 ± 0.4	3.1 ± 0.4	<.001
Stress, mean ± SD	12.7 ± 4.5	–	–	–	–	11.3 ± 4.1	15.8 ± 3.9	<.001
FCoV-19S, mean ± SD	17.1 ± 4.9	–	–	–	–	16.4 ± 4.7	19.1 ± 4.9	<.001
Depression			<.001		<.001			
No (PH*Q* < 2)	292 (70.7)	11.3 ± 4.1		16.4 ± 4.7		-	–	-
Yes (PH*Q* ≥ 2)	121 (29.3)	15.8 ± 3.9		19.1 ± 4.9		-	–	-

S-COVID-19-S: symptoms like COVID-19; FCoV-19S: fear of COVID-19; PHQ: Patient Health Questionnaire; HL: health literacy; SD: standard deviation.

*Results of the Chi-squared test for PHQ, and the *t*-test or one-way ANOVA test for stress and FCoV-19S.

### 3.2. Associated factors of stress

In bivariate models, the determinants of stress were weekly working hour changes, health status, health-related activity limitations, S-COVID-19-S, level of informing on COVID-19-related updates, confusion about COVID-19-related information, level of concern about getting infected, SoC and health literacy. We found that health-related activity limitations were correlated with health status at *rho* = −0.43, information confusion was correlated with informing on COVID-19 updates at *rho* = −0.32 (Table S1). Therefore, health-related activity limitations and confusion about COVID-19-related information were analysed in the multivariate model together with other factors that showed a significant association with stress in the bivariate model. The results show that factors associated with higher stress scores were health-related activity limitations (unstandardized regression coefficient, *B* = 0.92; 95% confidence interval, 95% CI = 0.05 to 1.79; *p* = .039), S-COVID-19-S (*B* = 1.52; 95%CI = 0.70 to 2.34; *p* < .001), COVID-19-related information confusion (*B* = 2.47; 95% CI = 1.15 to 3.79; *p* < .001). Conversely, sense of coherence (B = −1.39; 95%CI = −1.76 to −1.03; *p* < .001) and coronavirus-related HL (B = −1.76; 95% CI = −2.66 to −0.85; *p* < .001) were associated with lower stress scores (Table 2). The adjusted R^2^ = 0.266.

**Table 2.** Factors associated with stress in school principals (*n* = 413).

Variables	Stress			
	Bivariate		Multivariate	
	B (95% CI)	*p*	B (95% CI)	*p*
Age, years	−0.02 (−0.11, 0.07)	.605	−0.05 (−0.13, 0.03)	.208
Gender				
Women	0.00		0.00	
Men	−0.62 (−1.53, 0.30)	.188	−0.45 (−1.27, 0.38)	.286
School type				
Primary school	0.00		0.00	
Junior high school	0.53 (−0.58, 1.65)	.348	−0.25 (−1.25, 0.76)	.632
Senior high school or vocational school	−0.18 (−1.46, 1.10)	.782	0.04 (−1.11, 1.19)	.948
School for special children	−1.37 (−4.40, 1.65)	.372	−1.63 (−4.26, 1.00)	.225
School geography				
North	0.00		0.00	
Centre	0.29 (−0.82, 1.40)	.606	0.11 (−0.87, 1.09)	.825
South	0.68 (−0.58, 1.94)	.289	0.54 (−0.59, 1.66)	.351
East	0.37 (−1.21, 1.95)	.648	−0.18 (−1.58, 1.22)	.797
Outlying islands	0.71 (−1.19, 2.60)	.464	1.41 (−0.36, 3.18)	.118
School Size				
≤ 12 classes	0.00		–	–
13-24 classes	−0.51 (−1.76, 0.74)	.424	–	–
25-48 classes	−0.01 (−1.14, 1.12)	.986	–	–
≥ 49 classes	−0.07 (−1.31, 1.17)	.912	–	–
Involving teaching				
No	0.00		0.00	
Yes	−0.63 (−1.62, 0.36)	.211	−0.50 (−1.39, 0.39)	.270
Weekly working hours				
< 40 h	1.22 (−0.67, 3.12)	.206	0.88 (−0.80, 2.56)	.304
= 40 h	0.00		0.00	
> 40 h	0.13 (−1.11, 1.36)	.842	−0.09 (−1.20, 1.03)	.877
Weekly working hour changes				
Less than before the pandemic	0.41 (−0.60, 1.43)	.421	0.34 (−0.56, 1.24)	.460
About the same				
More than before the pandemic	1.36 (0.16, 2.56)	.027	0.86 (−0.19, 1.92)	.109
Health status				
Very bad/bad/moderate	0.00		–	–
Good/very good	−1.96 (−2.83, −1.08)	<.001	–	–
Chronic health conditions				
None	0.00		–	–
One or more	0.39 (−0.51, 1.29)	.395	–	–
Health-related activity limitations				
Not limited	0.00		0.00	
Limited	1.85 (0.90, 2.80)	<.001	0.92 (0.05, 1.79)	.039
S-COVID-19-S				
No	0.00		0.00	
Yes	1.86 (0.97, 2.75)	<.001	1.52 (0.70, 2.34)	<.001
COVID-19 vaccination				
No	0.00		0.00	
Yes	0.45 (−0.89, 1.78)	.511	0.01 (−1.26, 1.29)	.983
Level of informing on COVID-19 updates				
Insufficient/poor/fine informed	0.00		–	–
Well/very well informed	−2.95 (−4.26, −1.63)	<.001	–	–
Confusion about COVID-19-related information				
Not at all/little confused	0.00		0.00	
Quite/very confused	3.86 (2.44, 5.27)	<.001	2.47 (1.15, 3.79)	<.001
Level of concern about getting infected				
Slightly concerned/not concerned at all	0.00		0.00	
Very concerned/concerned	1.01 (0.09, 1.93)	.031	0.37 (−0.44, 1.18)	.370
Perceived likelihood of getting infected				
Not likely/definitely not	0.00		–	–
Very likely/somewhat likely	0.59 (−0.30, 1.48)	.192	–	–
Sense of coherence	−1.65 (−2.03, −1.28)	<.001	−1.39 (−1.76, −1.03)	<.001
Coronavirus-related HL	−3.20 (−4.13, −2.27)	<.001	−1.76 (−2.66, −0.85)	<.001

S-COVID-19-S: symptoms like COVID-19; HL: health literacy; B: unstandardized regression coefficient.

### 3.3. Factors associated with fear of COVID-19

In the bivariate analysis, the determinants of fear of COVID-19 were school type, weekly working hour changes, health status, health-related activity limitations, S-COVID-19-S, informing on COVID-19 updates, confusion about COVID-19-related information, level of concern about getting infected, perceived likelihood of getting infected, sense of coherence and health literacy. We found that health-related activity limitations were correlated with health status at *rho* = −0.43, information confusion was correlated with informing on COVD-19 updates at *rho* = −0.32, and level of concern about getting infected was correlated with the perceived likelihood of getting infected at *rho* = 0.80 (Table S1). Therefore, health-related activity limitations, information confusion and concern about getting infected were analysed in the multivariate model together with other factors that showed a significant association with stress in the bivariate model. The results show that factors associated with higher fear of COVID-19 scores were longer working hours during the pandemic (*B* = 1.69; 95% CI = 0.49 to 2.89; *p* = .006), COVID-19-related information confusion (*B* = 3.77; 95% CI = 2.20 to 5.34; *p* < .001) and concern about getting infected (*B* = 3.18; 95% CI = 2.27 to 4.08; *p* < .001). Conversely, higher coronavirus-related HL was associated with lower fear scores (B = −1.85; 95%CI = −2.90 to −0.81; *p* < .001) (Table 3). The adjusted R^2^ = 0.235.

**Table 3.** Factors associated with fear of COVID-19 in school principals (*n* = 403).

Variables	FCoV-19S			
	Bivariate		Multivariate	
	B (95% CI)	*p*	B (95% CI)	*p*
Age, years	0.02 (−0.08, 0.12)	.693	0.02 (−0.07, 0.11)	.704
Gender				
Women	0.00		0.00	
Men	−0.20 (−1.22, 0.81)	.692	0.27 (−0.66, 1.19)	.573
School type				
Primary school	0.00		0.00	
Junior high school	0.06 (−1.16, 1.29)	.917	−0.35 (−1.49, 0.79)	.546
Senior high school and vocational school	−1.49 (−2.89, −0.09)	.037	−1.28 (−2.57, 0.00)	.051
School for special children	−1.26 (−4.54, 2.02)	.451	−1.61 (−4.54, 1.32)	.279
School geography				
North	0.00		0.00	
Centre	0.20 (−1.03, 1.42)	.750	0.04 (−1.06, 1.15)	.938
South	−0.72 (−2.10, 0.67)	.308	−0.67 (−1.93, 0.60)	.301
East	−0.40 (−2.15, 1.36)	.658	−0.81 (−2.41, 0.78)	.316
Outlying islands	−0.56 (−2.62, 1.51)	.597	0.41 (−1.57, 2.39)	.685
School Size				
≤ 12 classes	0.00		-	–
13-24 classes	−0.62 (−2.00, 0.76)	.377	-	–
25-48 classes	−0.06 (−1.31, 1.20)	.931	-	–
≥ 49 classes	−0.37 (−1.73, 0.98)	.588	-	–
Involving teaching				
No	0.00		0.00	
Yes	−0.31 (−1.41, 0.78)	.574	−0.37 (−1.38, 0.63)	.467
Weekly working hours				
< 40 h	1.45 (−0.64, 3.53)	.174	1.26 (−0.62, 3.15)	.188
= 40 h	0.00		0.00	
> 40 h	0.17 (−1.18, 1.52)	.803	0.47 (−0.78, 1.72)	.457
Weekly working hour changes				
Less than before the pandemic	0.20 (−0.91, 1.31)	.718	0.28 (−0.73, 1.28)	.588
About the same	0.00		0.00	
More than before the pandemic	1.52 (0.18, 2.86)	.027	1.69 (0.49, 2.89)	.006
Health status				
Very bad/bad/moderate	0.00		-	–
Good/very good	−1.86 (−2.84, −0.89)	<.001	-	–
Chronic health conditions				
None	0.00		-	–
One or more	0.73 (−0.26, 1.72)	.148	-	–
Health-related activity limitations				
Not limited	0.00		0.00	
Limited	1.73 (0.67, 2.79)	.001	0.94 (−0.04, 1.93)	.060
S-COVID-19-S				
No	0.00		0.00	
Yes	1.35 (0.35, 2.35)	.008	0.93 (0.00, 1.85)	.051
COVID-19 vaccination				
No	0.00		0.00	
Yes	0.26 (−1.22, 1.74)	.727	−0.16 (−1.61, 1.29)	.829
Level of informing on COVID-19 updates				
Insufficient/poor/fine informed	0.00		-	–
Well/very well informed	−2.66 (−4.13, −1.19)	<.001	-	–
Confusion about COVID-19-related information				
Not at all/little confused	0.00		0.00	
Quite/very confused	4.67 (3.01, 6.32)	<.001	3.77 (2.20, 5.34)	<.001
Level of concern about getting infected				
Slightly concerned/not concerned at all	0.00		0.00	
Very concerned/concerned	3.66 (2.71, 4.61)	<.001	3.18 (2.27, 4.08)	<.001
Perceived likelihood of getting infected				
Not likely/definitely not	0.00		-	–
Very likely/somewhat likely	2.85 (1.92, 3.79)	<.001	-	–
Sense of coherence	−0.56 (−1.01, −0.11)	.016	−0.19 (−0.61, 0.22)	.364
Coronavirus-related HL	−3.15 (−4.22, −2.07)	<.001	−1.85 (−2.90, −0.81)	.001

S-COVID-19-S: symptoms like COVID-19; FCoV-19S: fear of COVID-19; HL: health literacy; B: unstandardized regression coefficient.

### 3.4. Factors associated with depression

In bivariate models, the determinants of depressive symptoms were age, school type, school location, school size, weekly working hour changes, health status, chronic health condition, health-related activity limitations, S-COVID-19-S, confusion about COVID-19-related information, sense of coherence and health literacy. We found that school location was correlated with school size at *rho* = −0.40, health-related activity limitations were correlated with health status at *rho* = −0.43, with chronic health conditions at *rho* = 0.55 (Table S1). Therefore, health-related activity limitations, confusion about COVID-19-related information and concern about getting infected and other factors significantly linked to depression in bivariate models were analysed in the multivariate model. The results show that factors associated with higher odds of depressive symptoms were health-related activity limitations (odds ratio, OR = 3.38; 95% CI = 2.02 to 5.66; *p* < .001), S-COVID-19-S (OR = 3.06; 95% CI = 1.84 to 5.07; *p* < .001). Conversely, age (OR = 0.92; 95% CI = 0.87 to 0.97; *p* = .003), sense of coherence (OR = 0.76; 95% CI = 0.60 to 0.96; *p* = .020) and coronavirus-related HL (OR = 0.53; 95% CI = 0.28 to 0.98; *p* = .043) were associated with a lower likelihood of depressive symptoms (Table 4).

**Table 4.** Factors associated with depression in school principals (*n* = 413).

Variables	Depression			
	Bivariate		Multivariate	
	OR (95% CI)	*P*	OR (95% CI)	*p*
Age, years	0.95 (0.91, 0.99)	.019	0.92 (0.87, 0.97)	.003
Gender				
Women	1.00		1.00	
Men	0.80 (0.51, 1.23)	.307	0.65 (0.38, 1.10)	.107
School type				
Primary school	1.00		1.00	
Junior high school	1.79 (1.07, 3.00)	.027	1.56 (0.84, 2.88)	.161
Senior high school or vocational school	1.01 (0.53, 1.90)	.984	1.14 (0.53, 2.42)	.739
School for special children	1.38 (0.34, 5.69)	.652	1.71 (0.30, 9.77)	.546
School location				
North	1.00		1.00	
Centre	1.75 (1.01, 3.02)	.045	1.70 (0.89, 3.21)	.106
South	1.55 (0.84, 2.88)	.164	1.56 (0.75, 3.23)	.232
East	1.13 (0.51, 2.51)	.771	1.15 (0.47, 2.82)	.758
Outlying islands	1.15 (0.44, 2.98)	.779	1.16 (0.35, 3.84)	.803
School Size				
≤ 12 classes	1.00		–	–
13-24 classes	0.47 (0.24, 0.92)	.027	–	–
25-48 classes	0.98 (0.58, 1.67)	.940	–	–
≥ 49 classes	0.89 (0.49, 1.60)	.686	–	–
Involving teaching				
No	1.00		1.00	
Yes	1.03 (0.64, 1.66)	.907	1.34 (0.74, 2.41)	.336
Weekly working hours				
< 40 h	1.64 (0.67, 3.99)	.276	2.35 (0.79, 6.96)	.122
= 40 h	1.00		1.00	
> 40 h	1.07 (0.58, 1.96)	.837	1.15 (0.54, 2.42)	.719
Weekly working hour changes				
Less than before the pandemic	1.17 (0.71, 1.93)	.532	1.20 (0.67, 2.15)	.533
About the same	1.00		1.00	
More than before the pandemic	1.93 (1.10, 3.37)	.021	1.76 (0.92, 3.37)	.090
Health status				
Very bad or bad or moderate	1.00		–	–
Good or very good	0.22 (0.14, 0.35)	<.001	–	–
Chronic health conditions				
No	1.00		–	–
Yes	1.98 (1.29, 3.05)	.002	–	–
Health-related activity limitations				
Not limited	1.00		1.00	
Limited	3.88 (2.46, 6.13)	<.001	3.38 (2.02, 5.66)	<.001
S-COVID-19-S				
No	1.00		1.00	
Yes	3.40 (2.19, 5.28)	<.001	3.06 (1.84, 5.07)	<.001
COVID-19 vaccination				
No	1.00		1.00	
Yes	1.24 (0.66, 2.32)	.499	1.11 (0.51, 2.41)	.791
Level of informing on COVID-19 updates				
Insufficient/poor/fine informed	1.00		–	–
Well or very well informed	0.86 (0.46, 1.63)	.655	–	–
Confusion about COVID-19-related information				
Not at all/little confused	1.00		1.00	
Quite/very confused	2.56 (1.33, 4.91)	.005	1.89 (0.86, 4.14)	.111
Level of concern about getting infected				
Slightly concerned/not concerned at all	1.00		1.00	
Very concerned/concerned	1.35 (0.85, 2.12)	.203	1.04 (0.61, 1.76)	.889
Perceived likelihood of getting infected				
Not likely/definitely not	1.00		–	–
Very likely/somewhat likely	1.14 (0.74, 1.75)	.556	–	–
Sense of coherence	0.73 (0.60, 0.89)	.002	0.76 (0.60, 0.96)	.020
Coronavirus-related HL	0.39 (0.23, 0.64)	<.001	0.53 (0.28, 0.98)	.043

S-COVID-19-S, symptoms like COVID-19; PHQ, Patient Health Questionnaire; HL, health literacy; OR, odds ratio.

## 4. Discussion

This study aimed to explore the associated factors of stress, FCoV-19S and depressive symptoms among school principals. We found that school principals with S-COVID-19-S had higher levels of stress and a higher likelihood of having depressive symptoms than their counterparts. This finding was consistent with previous studies conducted on various populations, including healthcare workers [26] and patients [22,37]. During the difficult pandemic time, school principals have to deal with additional work to ensure that teaching and learning activities are maintained. These tasks may be affected if school heads become infected with COVID-19. Thus, S-COVID-19-S raises their concerns about their health and ability to accomplish work. As a result, school leaders with S-COVID-19-S may experience more pressure and stress, leading to psychological problems (e.g. depression). Our results also indicated that school principals with health-related activity limitations were more likely to have depression and a higher stress score. The positive association of physical limitations with stress and depression was documented in previous research among different populations [69–71]. An explanation could be that health-related activity limitations may reduce social connections and recreational activities, leading to isolation and prolonged stress [72]. These factors are significant predictors of depression. From these findings, occupational health surveillance should be promoted to monitor the mental and physical health conditions of school principals and teachers during the COVID-19 pandemic [73]. Furthermore, workplace health improvement activities should be promoted to prevent the onset of long-term consequences for the mental health of school principals and teachers [74,75].

Moreover, this study indicated that a higher work-related sense of coherence was linked to a lower stress score and a lower likelihood of depressive symptoms. These results were in line with prior research conducted on various populations and professions, including adolescents [76,77], adults [78], high school teachers [79], software workers [80] and nurses [81]. Sense of coherence (SOC) is reflected through people's capacity to cope with daily life stressors and is characterized by comprehensibility, manageability and meaningfulness [40]. Thus, during the pandemic, having a higher SOC score could enable school leaders to better understand stressful situations and available resources to manage, control, arrange work properly and make healthier decisions. These factors may help reduce pressure and psychological problems.

The current study also indicated that school principals who had a longer working time than before the pandemic had a higher score of COVID-19-related fear. The explanation for this association is that increased workload can lead to fatigue, tension and emotional exhaustion [82,83]. The pressure from working longer hours can also lead to health issues (e.g. high blood pressure, weight gain [17], sleep disorders [13], breathing problems and headaches [16]). As a result, when school principals are emotionally and physically exhausted, they may have negative thoughts about their vulnerability to COVID-19, reducing their sense of safety and increasing fear. In addition, we found that school leaders concerning about getting COVID-19 had higher fear scores. This relationship could be explained by the uncertainty of the pandemic and no specific treatment for COVID-19 [1]. Thus, being COVID-19 infected could pose risks to them and their loved ones such as death, isolation and post COVID-19 complications, thereby increasing their feeling of fear.

Our findings showed that participants who felt confused about the COVID-19-related information had a higher score of fear and stress. The results were consistent with a previous study conducted in China that suggested the information uncertainty about COVID-19 was positively associated with psychological stress [84]. During the COVID-19 crisis, many inaccurate information and conspiracy theories were disseminated broadly on social networks, raising concerns about the severity of COVID-19 and the effectiveness of prevention efforts [85–87]. As a result, when facing the uncertainty and ambiguity of the pandemic, people would feel more fearful. In addition, due to the confusion of COVID-19-related information along with the frequently changing prevention regulations, it may be more difficult for school administrators to make decisions in managing and sustaining a safe learning environment during the pandemic. This factor may lead to increased stress at work. Therefore, health organizations and government agencies should provide as accurate, transparent, and timely information as possible about the pandemic to help raise knowledge about COVID-19 (e.g. how it is transmitted or how to prevent it), thereby reducing fear and tension for the community, including school principals.

We also found that a higher COVID-19-related HL score was associated with a lower score of fear and stress, and a lower depression likelihood. During the pandemic, the benefit of HL in mitigating fear and psychological problems was documented in recent literature [28,37,88]. Improving HL could help to prevent school leaders from fake and misleading information during the infodemic [89], thereby reducing confusion and anxiety [32,90]. In addition, higher HL was found to be associated with better adherence to protective behaviours (e.g. handwashing, mask-wearing, physical distancing) [91,92] and healthier lifestyles (e.g. healthy eating, keeping physically active) [93,94], which could help them to alleviate fear and improve psychological health during the pandemic. Moreover, finding from Germany could show that individual health literacy of school principals was positively associated with the implementation of holistic activities in school health promotion [90]. Hence, investing in strengthening health literacy at an individual level might also empower school leaders to support health-promoting activities in their schools with potentially positive effects on students and school staff. Our study showed that older age was associated with a lower likelihood of depressive symptoms. However, previous studies showed inconsistent results on this association. A study in Norway indicated that advanced age was positively associated with the prevalence of depression among people aged 20–89 years [95]. Another study conducted on individuals aged 53–80 years in Germany showed people older than 60 were less likely to have depression than those aged 53–59 [96]. A possible explanation for our finding is that principals with a higher age may have more experience in managing and solving difficulties at work. As a result, when faced with unexpected and challenging situations in the pandemic, they could better organize their work and make more proper decisions, thereby reducing stress and mental problems.

This study has several limitations. First, since it was a cross-sectional study, a cause-and-effect relationship could not be drawn from this study. Next, we used a network sampling method with a low response rate of about 10.5%, which may influence the generalizability of our findings. Finally, several confounders that may affect the results, such as lifestyles, and coping behaviours, were not investigated in this study. Future research should examine more potential factors and associations. However, our study could provide timely evidence for proper strategies to improve the mental health of school principals who play a major part in recovering schools and education systems from the adverse impact of the pandemic.

## 5. Conclusions

During the pandemic, school principals with health-related activity limitations and S-COVID-19-S were more likely to have stress and depression, whereas those with a higher sense of coherence were less like to have stress and depression. Better health literacy was inversely associated with FCoV-19S, stress and depressive symptoms, whereas COVID-19-related information confusion had a positive association with FCoV-19S and stress. In addition, school principals with a longer working time than before the pandemic were more likely to have higher FCoV-19S. Our study provides evidence to develop appropriate actions to mitigate fear, stress and psychological problems for school principals.

## Supplementary Material

Supplemental Material

## Data Availability

The data that support the findings of this study are available from the corresponding author upon reasonable request.
